# GC-MS-Based Metabolomics to Reveal the Protective Effect of Gross Saponins of *Tribulus terrestris* Fruit against Ischemic Stroke in Rat

**DOI:** 10.3390/molecules24040793

**Published:** 2019-02-22

**Authors:** Yang Wang, Hongyu Zhao, Yue Liu, Wenjun Guo, Yanru Bao, Manqi Zhang, Tunhai Xu, Shengxu Xie, Xinyu Liu, Yajuan Xu

**Affiliations:** 1 Jilin Ginseng Academy, Changchun University of Chinese Medicine, Changchun 130117, China; white-wing@163.com; 2 Key Laboratory of Medicinal Materials, Jilin Academy of Chinese Medicine Sciences, Changchun 130021, China; fixiov5815@126.com (H.Z.); yue_liu_tryh@126.com (Y.L.); 19969540912@163.com (W.G.); 024baoyanru@163.com (Y.B.); mzhan016@fiu.edu (M.Z.); jshx222@126.com (S.X.); liuxinyu6868@126.com (X.L.); 3 Academy of Traditional Chinese Medicine, Beijing University of Chinese Medicine, Beijing 100029, China

**Keywords:** GC-MS, metabolomics, gross saponins of *Tribulus terrestris*, ischemic stroke

## Abstract

Stroke is one of the most common neurological disorders and seriously threatens human life. Gross saponins of *Tribulus terrestris* fruit (GSTTF) are used for neuroprotective treatment on convalescents of ischemic stroke. However, the therapeutic effects and mechanisms have not yet well understood, especially from the metabolic perspective. In this study, the protective effect of GSTTF on ischemic stroke in a middle cerebral artery occlusion (MCAO) rat model was investigated by the GC-MS-based metabolomics approach. 2,3,5-triphenyltetrazolium chloride (TTC) staining of brain tissues showed that GSTTF significantly reduced the infarct area after MCAO surgery. Metabolomic profiling showed a series of metabolic perturbation occurs in ischemic stroke compared with sham group. GSTTF can reverse the MCAO-induced serum metabolic deviations by regulating multiple metabolic pathways including fatty acids metabolism, amino acids metabolism, and carbohydrates metabolism. The current study provided a useful approach for understanding the mechanism of MCAO-induced ischemic stroke and a reliable basis for evaluating the efficacy of GSTTF in the treatment of ischemic stroke.

## 1. Introduction

Stroke is one of the most fatal neurological diseases, the second leading cause of death in people over 60 years old and the fifth leading cause of death in people aged 15–59 worldwide [1]. More than 80% of all strokes are caused by ischemic events, resulting in devastating neurological sequelae accompanied by severe morphological and molecular alterations [2]. The mechanisms and methods for precise pathological of ischemic brain injury has not yet mature; there are no generally accepted and effective treatments for ischemic stroke. Currently, the first-choice treatment for ischemic stroke is thrombolysis, which attempts to dissolve the clots, restore blood flow, and preserve the surrounding brain tissue. However, this strategy could change the course of ischemia only if performed in a narrow time window [3,4].

Traditional Chinese medicine (TCM) has been practiced to treat disease and disorders for thousands of years in China. Herbal medicine, as well as the formulae, have been reported to treat cerebral ischemia [5,6,7]. *Tribulus terrestris* is an annual creeping herb widespread in China, India, Japan, and Korea. Its fruit has been used in traditional Chinese medicine for the treatment of eye disease, edema, abdominal distention, sexual dysfunction, high blood pressure, and cardiovascular diseases. The gross saponins of *Tribulus terrestris* fruit (GSTTF) are a mixture of dozens of different types of saponins, mainly steroid saponins. Food supplements containing *Tribulus terrestris* extracts are on sale in USA and Europe with the claim of a general stimulating action on motor activity, muscle tone and restorative tonic for vigour [8]. Although previous studies showed that gross saponins of *Tribulus terrestris* could protect the brain from ischemic injury in vivo and in vitro, little is known about its effects from metabolic perspective [9,10].

Metabolomics is an emerging platform of system biology to profile entire endogenous metabolites in biological systems and to monitor their fluctuations related to genetic, biological, or environmental perturbation. The analysis of metabolites can provide global changes of end products in the body, and has been widely used in biomarker discovery, toxicity evaluation and drug efficacy assessments [11,12,13]. The global analysis of metabolite for metabolomics is consistent with the holistic thinking of TCM and the multiple pharmacological activities of herbal medicine, making metabolomics suitable for understanding disease pathogenesis and the therapeutic mechanism of herbal medicine at the metabolic level.

In this study, an MCAO rat model with ischemic stroke was constructed and injected with GSTTF via tail vein injection. The changes in the serum metabolome were determined by GC-MS-based metabolomics to investigate the protective mechanisms of GSTTF during the pathogenesis of ischemic stroke. The potential serum biomarkers and the related perturbed metabolic pathways of MCAO were identified. This study provides a reference for the understanding of the pathogenesis of ischemic stroke and the therapeutic mechanisms of GSTTF.

## 2. Results

### 2.1. The Effects of GSTTF on MACO in Rats

**Figure 1:**
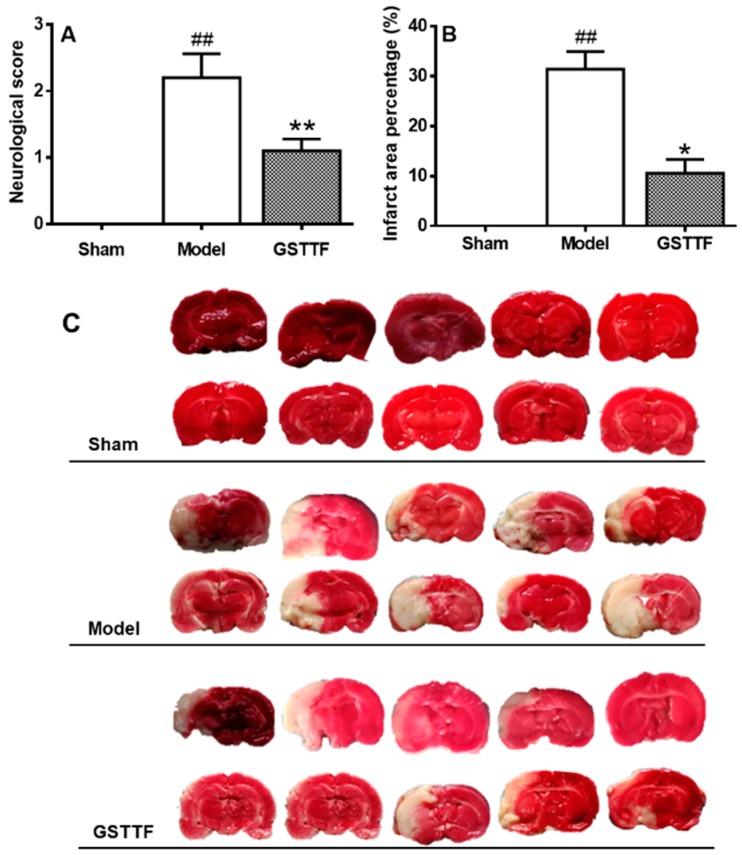
Effects of GSTTF on MCAO rats, neurobehavioral score (**A**), infarct area (**B**) and TTC staining of brain (**C**). ^##^ *p* < 0.01, the model group versus the sham group; * *p* < 0.05, ** *p* < 0.01, the GSTTF-treated group versus the model group.

Neurologic score and infarct volume measurement were performed to evaluate the animal model. As shown in Figure 1A, the rat from sham group undergone sham-operated surgery, therefore, they did not have any neurological defect and had the score of zero. The rats in the model group obtain the highest score among the three groups, indicating a neurological defect after MCAO. After treated with GSTTF, the neurological defect was significantly improved compared to the model group (from 2.20 ± 1.14 to 1.10 ± 0.57).

Infarct volume of each group was shown in Figure 1B,C, the rat from sham group had no cerebral injury, thus, no infarct area was observed. The MCAO surgery yielded a remarkable infarct area in the coronal brain sections, which is consistent with that they had the highest neurological defect score among the groups. In GSTTF-treated group, a significant recovery (*p* < 0.01) in the infarct area was observed in TTC stained cerebral slices. The results demonstrate that the administration of GSTTF prior to MCAO had a neuroprotective effect in rats.

### 2.2. GC-TOF/MS Method Validation

**Figure 2:**
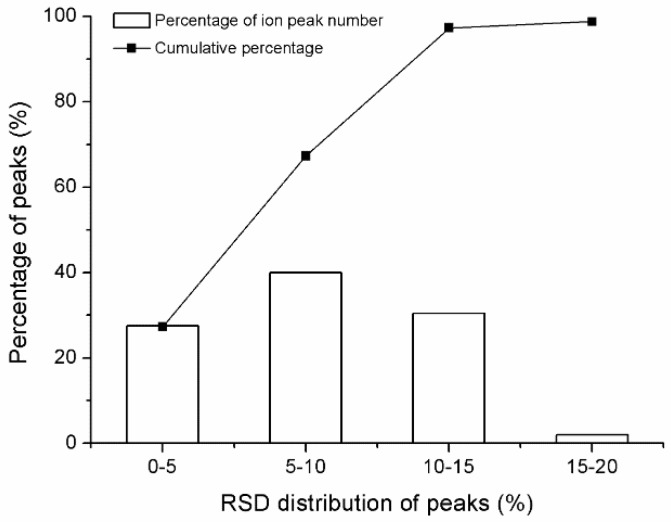
RSD (%) distribution of all metabolites in the pooled quality control (QC) samples.

The reproducibility and stability of the GC-TOF/MS method were essential in metabolomics analysis and was evaluated by using the QC samples. During the analytical run, five injections of QC sample were performed initially and then one injection was analyzed every fourth analytical run to provide robust quality assurance for each metabolic feature. A total of 435 peaks were acquired from serum sample. After alignment and normalization, ions that were detected in <50% of the QC samples were removed from the dataset, and 379 ion peaks were reproducibly detected from the raw GC-TOF/MS chromatogram of the QC samples. The repeatability of peak intensities of 379 ions detected from QC sample was evaluated and expressed by the relative standard deviation (RSD) distribution. As shown in Figure 2, 27.5%, 67.5%, and 97.5% of the peaks had RSD values lower than 5, 10, and 15%, respectively, which illustrate that the repeatability and stability of the developed method were suitable for metabolic profiling analysis.

### 2.3. Metabolic Profile in Serum

The serum extracts were analyzed by the validated GC-QTOF/MS method, the typical total ion chromatogram (TIC) of three groups were shown in Figure S1 (supplementary material). Good separation was achieved in 25 min under the established analytical method, and subtle differences among these groups were observed, but the visual inspection was not enough to elucidate the metabolic differences between sham group and model group and further to evaluate the therapeutic effect of GSTTF. Therefore, multivariate statistical analysis was conducted to investigate the changes of serum metabolites in three groups.

**Figure 3:**
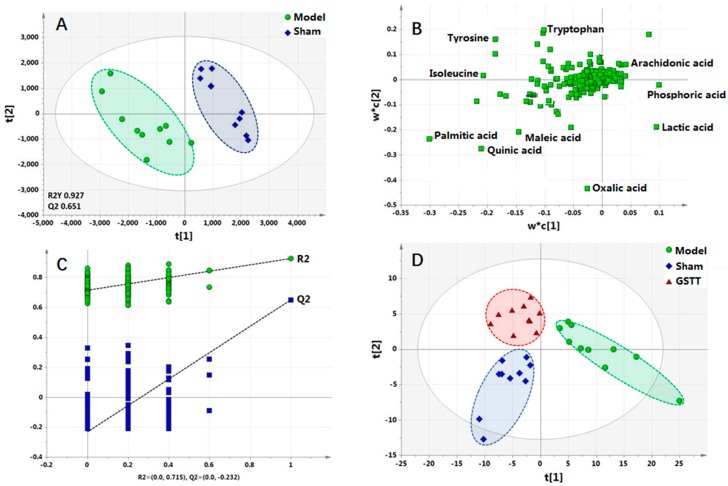
PLS-DA (**A**) score plot, (**B**) loading plot, (**C**) permutation test of sham and model groups, and (**D**) PLS-DA score plot of sham, model, and GSTTF-treated groups.

To make an overview on the differences in serum metabolic profiling between sham group and model group, the 379 peaks were used to construct partial least-squares discriminant analysis (PLS-DA) model with Pareto scaling. As shown in the score plot of PLS-DA (Figure 3A), clear separation between these two groups in the first principal component, which suggests the serum metabolic pattern has been altered after MACO surgery. The parameters R2Y and Q2 value of 0.927 and 0.651 were considered to be an excellent fitness and prediction ability of the established PLS-DA model. The permutation test (*n* = 200) was further conducted to validate the model and avoid overfitting. As shown in Figure 3C, the value of intercepts, R2 (0.715) and Q2 (−0.232), were lower than the original value, which indicate great predictability and goodness of fit of the established model.

The cluster of GSTTF-treated group was close to the sham group (Figure 3D) indicating that the protective effect of GSTTF to the MACO rats, which was consistent with the result of infarct measurement.

### 2.4. Biomarker Selection and Metabolic Pathway Analysis

The metabolites that contribute to the separation between the sham group and model group were revealed using variable importance in the projection (VIP) value, *p*-value and fold change value. In general, the variables with VIP1 and VIP 2 values were larger than 1 were highlighted as candidate biomarkers, then these variables were further filtered by fold change and Student’s t-test to select the features with a significant difference between two groups. Finally, a total of 41 highlighted metabolites with *p* < 0.05 and fold change >2 were kept as the potential biomarkers and labeled in the loading plot (Figure 3C). These significantly changed metabolites (Table 1) include 13 fatty acids, 10 amino acids, seven organic acids, six carbohydrates, and seven other metabolites. The detailed information and the representative mass spectra of the metabolites are shown in Table S1 and Figure S2.

**Figure 4:**
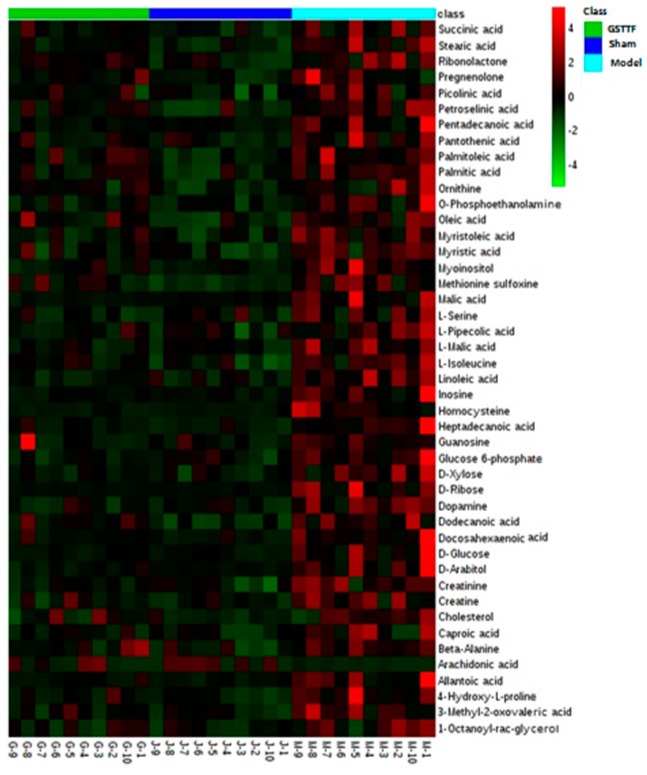
Heat map visualizing the changes in the contents of potential biomarkers. Rows: biomarkers; Columns: samples. Color key indicates the concentration of metabolites, green: lowest, red: highest.

To visualize the tendency of the variation of the biomarkers among three groups, a heat map was constructed based on the relative quantities of each metabolite. As shown in Figure 4, color differences in model group and sham group indicated the metabolic perturbation after MCAO surgery. Compared to the sham group, the content of most metabolites increased in the model group, including amino acids, fatty acids except arachidonic acid, and sugar alcohol, while after treated with GSTTF, the level of these metabolites was partly reduced and close to the level in sham group.

**Figure 5:**
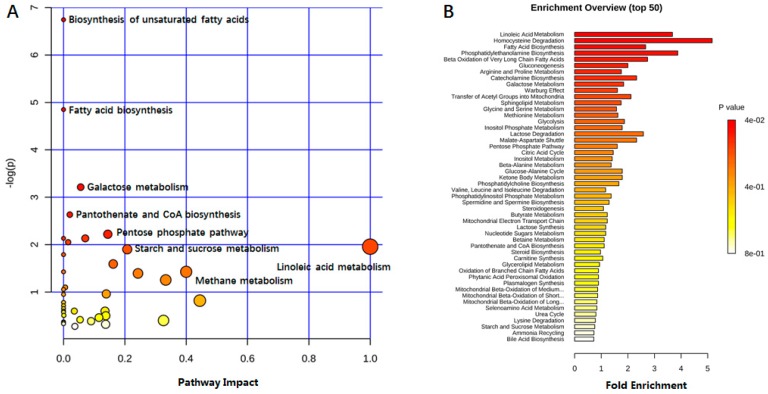
Altered metabolic pathway in MCAO rats as (**A**) visualized by bubble plots and (**B**) enrichment overview.

These biomarkers were further imported into MetaboAnalyst 4.0 software (McGill University, Montreal, QC, Canada) to perform enrichment analysis and pathway analysis (Figure 5). The significant relevant pathways influenced by MCAO surgery, including linoleic acid metabolism, homocysteine degradation, fatty acid biosynthesis, amino acid metabolism, etc., are highlighted.

## 3. Discussion

In this study, serum samples from model group, sham group and GSTTF-treated group were analyzed by GC-MS-based metabolomics method to reveal the significant different metabolite between sham group and model group, and to evaluate the protective effect and relative mechanism of GSTTF. The infarct volume in GSTTF-treated group yielded smaller infarct size than that in model group, suggesting the protective effect of GSTTF for ischemic stroke. Consistent with this result, PLS-DA analysis of GC-MS-based profiles showed that the cluster of GSTTF-treated group was separated with the model group and close to the sham group. A total of 41 endogenous metabolites were selected as biomarkers and their related pathways were further investigated.

Ischemic stroke results from a sudden blockage of an artery that provides blood to the brain. When an ischemic stroke occurs, the blood supply to the brain is interrupted, and the brain cells are unable to obtain sufficient oxygen or nutrients that they need to function. Therefore, the brain cells at the blockage site died and released toxic chemicals to damage the surrounding brain tissues [14,15]. Our untargeted GC-MS-based metabolomics analysis found the ischemic stroke greatly affected several metabolites that associate with fatty acid metabolism, amino acid metabolism, carbohydrate metabolism, and so on.

Fatty acids (FA) composition affects several different physiological and biochemical process, including glucose metabolism [16,17], lipid metabolism [18,19], blood pressure [20,21], platelet aggregation [22,23], etc. Several studies have examined the relations between serum fatty acids and ischemic stroke risk, but the results were not consistent. A Japanese study found that high serum saturated fatty acids (SFA) were associated with an increased risk of ischemic stroke, and high serum linoleic acid was associated with a decreased risk [24]. In a study of US cohort of whites, the author found similar associations as the Japanese study but additionally reported that significant positive associations of monounsaturated fatty acids (MUFA) with ischemic stroke [25]. And a Finland study reported that n3 polyunsaturated fatty acids (PUFA) were associated with increased risk of ischemic stroke [26], but no associations of ischemic stroke with n3 PUFA was observed in the cohort of white subjects [25]. In this study, we found that all SFA and MUFA highlighted as biomarkers were greatly increased in the serum of MCAO rats. For PUFA, linoleic and arachidonic acids were selected as biomarkers, and these two n6 PUFA have inverse relations with ischemic stroke.

Three possibilities have been proposed to explain these inconsistencies among studies [25,27]. First is that whether the studies were adjusted or not according to factors potentially on the causal pathway (e.g., hypertension and lipids); second is the associations between serum fatty acids and ischemic stroke may not be consistent for all ischemic stroke subtypes (large-artery occlusive, lacunar and hemorrhagic). The inconsistent findings could also be explained by the different effects of FA on ischemic stroke. Iso et al. [24] reported that high serum myristic and palmitic acids, but not stearic acid, could increase the risk of ischemic stroke, although all three are SFA. Several studies also showed that individual fatty acids, even those of the same type, may have different effects on ischemic stroke or cardiovascular disease risk [28,29,30]. For example, compared with oleic acid, myristic and palmitic acids could increase plasma total and low-density lipoprotein cholesterol concentrations, whereas stearic acid does not [31]. Therefore, studying the associations between individual FA and ischemic stroke is important.

Several biomarkers identified in this study were amino acids (AA), which were significantly increased in model group compared with that in sham group. They are the decomposition products of proteins, and the changes in the level of AA may serve as diagnostic biomarkers for stroke.

Numerous studies have demonstrated that the synthesis of proteins was inhibited, and the decomposition process was activated, which lead to increased amino acid levels in the blood [32,33,34,35]. Serine and alanine serve as fuel substrates in energy metabolism for brain cells to meet their high energy demands. These two AA can be converted to pyruvate through glycolysis for energy supply. Isoleucine is important energy sources via the tricarboxylic acid (TCA) cycle after transformation into acetyl-CoA. The reduction in these AA may be due to reduced utilization of downstream energy metabolism, which could affect brain function. Homocysteine is a sulfhydryl product of methionine metabolism, increased methionine can lead to a high level of homocysteine, thereby causing stroke [36,37].

Saponins are the main components of *Tribulus terrestris* fruit. As shown in Figure 3D, after treatment with GSTTF, the cluster of treated group was close to the sham group, suggesting the GSTTF have therapeutic effect on MCAO, which were confirmed by the infarct volume measurement and neurologic score. FA and AA are two main types of biomarkers, their levels were significantly altered by MCAO compared with sham group, and the GSTTF could partly recover the level of these metabolites. Thus, GSTTF has good potential to treat ischemic stroke in clinical applications.

## 4. Materials and Methods

### 4.1. Materials

Fatty acid methyl ester (C7–C30) standards and pyridine were purchased from Sigma-Aldrich (St. Louis, MO, USA). *N*-Methyl-*N*-trimethylsilyl-trifluoroacetamide (MSTFA) with 1% trimethylchlorosilane (TMCS) and chromatographic grade methanol were obtained from Thermo-Fisher Scientific (FairLawn, NJ, USA). Ultrapure water was prepared using a Milli-Q purifcation system (Billerica, MA, USA). The gross saponins of *Tribulus terrestris* fruit (purity of saponins: above 60%, Lot No. 0418303) was manufactured by Changbaishan Pharmaceutical Co. Ltd. (Jilin, China).

Retention index (RI) markers were prepared by dissolving fatty acid methyl esters (FAMEs) in hexane at a concentration of 1.6 mg/mL (C7–C16) and 0.8 mg/mL (C18–C30), respectively.

### 4.2. Animals and Treatments

A total of 30 adult male Sprague Dawley (SD) rats weighing 200 ± 20 g were purchased from the Beijing Vital River Laboratory Animal Technology Co., Ltd. (Beijing, China). Animals were maintained at room temperature (19–23 °C) with 12/12 h light/dark cycles at 40–65% relative humidity and fed by standard rat chow and water ad libitum. All animal treatments were approved by the Animal Ethics Committee, Academy of Traditional Chinese Medicine of Jilin Province (approval no. JLSZKYDWLL-2018-015).

After two weeks acclimatization, the total of 30 rats was divided randomly into three groups, including sham group, middle cerebral artery occlusion (MCAO) group, and gross saponin of *Tribulus terrestris* fruit (GSTTF)-treated group. The administration of GSTTF (3 mg/kg) was performed via tail vein injection for three days before and 24 h after MACO surgery for GSTTF-treated rats. The sham-operated and MCAO rats were administrated with the same volume of saline. All rats were anesthetized with 10% chloral hydrate (3 mL/kg body weight) before surgery. The MCAO surgery was performed as by suture ligation as previously reported with modifications [38,39]. Briefly, a 1 cm long skin midline incision was made on the neck, and the common carotid artery (CCA), the internal carotid artery (ICA) and the external carotid artery (ECA) were separated. Then a silicone coated suture was inserted from the left ECA into the lumen of the ICA to occlude the origin of the MCA. All animals were maintained at 25–28 °C during the surgery. Sham-operated animals were subjected to an identical procedure with the exception that MCA was not ligated.

### 4.3. Evaluation of Neurological Defects

Neurological functional deficiency scores were in accordance with Longa’s five-point scale [39]: zero points: no neurologic function damage; one point: failure to extend the contralateral front limb completely; two points: turning around to the other side gently or circling counter clockwise; three points: Turn around to the other side seriously; four points: cannot walk spontaneously. The higher the score, the more serious the impairment of animal behavior.

### 4.4. Infarct Volume Measurement

The rats were sacrificed by neck dislocation to access infarct outcome. Then brains were removed and sliced in 2-mm-thick coronal section and stained with 2,3,5-triphenyltetrazolium chloride (TTC, 2% TTC in phosphate-buffered saline). All coronal slices were digitalized, and the area of cerebral damage was analyzed using Image-J software (National Institutes of Health, Bethesda, MD, USA).

### 4.5. Sample Preparation

Serum samples were thawed on the ice at 4 °C and mixed thoroughly before preparation. Serum metabolites were extracted by adding 175 μL of pre-chilled methanol/chloroform (*v*/*v* = 3/1) solution and 10 μL internal standards to 50 μL of serum sample. After vortexing for 30 s, the samples were centrifuged at 14,000 g and 4 °C for 20 min, and the supernatant was transferred to a new centrifuge tube and lyophilized with a freeze dryer (Labconco, Kansas City, MO, USA).

The residue was derivatized by the previously reported procedure with modifications [40,41,42]: 50 μL of 20 mg/mL methoxyamine in pyridine was added to the dried residue to inhibit the ring formation of reducing sugars, protecting also all other aldehydes and ketones, vortexed and incubated at 30 °C for 2 h. After returning the samples at room temperature, 50 μL of MSTFA (1% TMCS) were added, vortexed and incubated at 37.5 °C for 1h for trimethylsilylation of acidic protons to increase the volatility of metabolites. FAMEs as RI markers were added to the solution to monitor the injection quality and perform automated retention time correction. Samples were then centrifuged at 14,000× *g* for 15 min, and the supernatant was used for GC-MS analysis.

Quality control (QC) samples were prepared by pooling volumes of all serum samples and were processed with the same procedure employed for the experiment samples.

### 4.6. GC-MS Analysis

A volume of 1 μL of derivatized sample was injected through a splitless injector into an Agilent 7890B gas chromatography-time-of-flight mass spectrometry (GC-TOF/MS) equipped with a Rxi-5 ms capillary column (30 m × 0.25 mm × 0.25 μm, Restek Corporation, Bellefonte, PA, USA). Helium was used as the carrier gas with a constant flow rate of 1 mL/min. The temperature program was as follows: the initial temperature was 80 °C hold for 2 min, elevated to 300 °C at a rate of 12 °C/min and was maintained for 4.5 min, and then increased to 320 °C at a rate of 40 °C/min for 1 min. The post-run is one minute to allow the oven to cool down to 80 °C. The injection temperature, transfer line, and ion source were set to 270, 270, and 220 °C, respectively, and the solvent delay time was set to 5 min. The mass range (*m*/*z* 39–550) in a full-scan mode for electron impact ionization (70 eV) was applied.

### 4.7. Data Processing and Statistical Analysis

Raw GC-TOF/MS data were processed using XploreMET software (v3.0 Metabo-Profile, Shanghai, China) for automated baseline denoising, peak detection, deconvolution, and signal alignment. The resulting data matrix containing variables, sample code, and peak area were exported as csv file and imported into SIMCA-P software (v13.0, Umetrics, Umeå, Sweden) to conduct multivariate statistical analysis. The Student’s t-test was used to assess the significant difference between groups using GraphPad Prism 6.0 (GraphPad Software, La Jolla, CA, USA). The altered pathways were determined with an open source, web-based software, MetaboAnalyst 4.0 (http://www.metaboanalyst.ca).

Metabolite annotation was performed by comparing the RIs and mass spectral data with JiaLib metabolite database that consist of 1200 mammalian metabolites with 15-year accumulation [43,44,45].

## 5. Conclusions

In this study, the protective effect of GSTTF on ischemic stroke was investigated by the GC-MS-based metabolomics. TTC staining of brain tissues showed that GSTTF greatly reduced the infarct area after MCAO. Subsequent metabolomics analysis showed different serum metabolic profile among sham, model and GSTTF-treated groups. A total of 41 metabolites in serum were identified as biomarkers and the related pathways analysis were further conducted. It is notable that several fatty acids and amino acids were highlighted as the differential metabolites in response to the ischemic stroke, indicating the connection between ischemic strokes and fatty acids or amino acids metabolism. After GSTTF treatment, the decreases in neurological defect and infarct size were observed, and the levels of biomarkers showed obvious trends toward to that in sham group. These results suggest the GSTTF exerted an effect on serum metabolome in MCAO-induced ischemic rats. The mechanism of GSTTF in neuroprotective treatment will be further investigated in future study. However, all metabolites were only detected by a single technique of GC-MS, which is a limitation for the biomarker discovery. Therefore, further analysis combined with LC-MS should be taken into account.

## Figures and Tables

**Table 1:** Identified endogenous metabolites and their change trends in serum.

No.	Metabolites	Formula	HMDB ID	Classification	*p*-Value	
					Model vs. Sham	GSTTF vs. Model
1	4-Hydroxy-proline	C_5_H_9_NO_3_	06055	Amino Acid	↑ ^##^	↓ *
2	Allantoic acid	C_4_H_8_N_4_O_4_	01209	Purine Derivative	↑ ^#^	↓ *
3	Beta-Alanine	C_3_H_7_NO_2_	00056	Amino Acid	↑ ^##^	↓
4	Creatine	C_4_H_9_N_3_O_2_	00064	Amino Acid	↑ ^##^	↓ *
5	Creatinine	C_4_H_7_N_3_O	00562	Amino Acid	↑ ^##^	↓ **
6	Homocysteine	C_4_H_9_NO_2_S	00742	Amino Acid	↑ ^##^	↓ **
7	Isoleucine	C_6_H_13_NO_2_	00172	Amino Acid	↑ ^###^	↓ **
8	Serine	C3H_7_NO_3_	00187	Amino Acid	↑ ^#^	↓ *
9	Methionine sulfoxide	C_5_H_11_NO_3_S	02005	Amino Acid	↑ ^##^	↓
10	Ornithine	C_5_H_12_N_2_O_2_	00214	Amino Acid	↑ ^###^	↓
11	Arabitol	C_5_H_12_O_5_	00568	Carbohydrates	↑ ^#^	↓ *
12	Glucose	C_6_H_12_O_6_	00122	Carbohydrates	↑ ^#^	↓ *
13	Ribose	C_5_H_10_O_5_	00283	Carbohydrates	↑ ^#^	↓ *
14	Xylose	C_5_H_10_O_5_	00098	Carbohydrates	↑ ^##^	↓ **
15	Glucose 6-phosphate	C_6_H_13_O_9_P	01401	Carbohydrates	↑ ^#^	↓ **
16	Ribonolactone	C_5_H_8_O_5_	01900	Carbohydrates	↑ ^##^	↓
17	Arachidonic acid	C_20_H_32_O_2_	01043	Fatty Acids	↓ ^###^	↑ ***
18	Caproic acid	C_6_H_12_O_2_	00535	Fatty Acids	↑ ^#^	↓
19	Docosahexaenoic acid	C_22_H_32_O_2_	02183	Fatty Acids	↑ ^#^	↓
20	Dodecanoic acid	C_12_H_24_O_2_	00638	Fatty Acids	↑ ^##^	↓
21	Heptadecanoic acid	C_17_H_34_O_2_	02259	Fatty Acids	↑ ^#^	↓ *
22	Linoleic acid	C_18_H_32_O_2_	00673	Fatty Acids	↑ ^##^	↓ ***
23	Myristic acid	C_14_H_28_O_2_	00806	Fatty Acids	↑ ^##^	↓ *
24	Myristoleic acid	C_14_H_26_O_2_	02000	Fatty Acids	↑ ^###^	↓ **
25	Oleic acid	C_18_H_34_O_2_	00207	Fatty Acids	↑ ^#^	↓
26	Palmitic acid	C_16_H_32_O_2_	00220	Fatty Acids	↑ ^##^	↓ *
27	Palmitoleic acid	C_16_H_30_O_2_	03229	Fatty Acids	↑ ^#^	↓
28	Pentadecanoic acid	C_15_H_30_O_2_	00826	Fatty Acids	↑ ^##^	↓ *
29	Stearic acid	C_18_H_36_O_2_	00827	Fatty Acids	↑ ^#^	↓ **
30	Cholesterol	C27H46O	00067	Lipids	↑ ^##^	↓
31	*O*-Phosphoethanolamine	C_2_H_8_NO_4_P	00224	Lipids	↑ ^#^	↓ *
32	Pregnenolone	C_21_H_32_O_2_	00253	Lipids	↑ ^#^	↓
33	Guanosine	C_10_H_13_N_5_O_5_	00133	Nucleotide	↑ ^##^	↓
34	Inosine	C_10_H_12_N_4_O_5_	00195	Nucleotide	↑ ^#^	↓ *
35	Pipecolic acid	C_6_H_11_NO_2_	00716	Organic Acids	↑ ^#^	↓ *
36	Malic acid	C_4_H_6_O_5_	00744	Organic Acids	↑ ^###^	↓ *
37	Petroselinic acid	C_18_H_34_O_2_	02080	Organic Acids	↑ ^##^	↓
48	Picolinic acid	C_6_H_5_NO_2_	02243	Organic Acids	↑ ^#^	↓ *
49	Succinic acid	C_4_H_6_O_4_	00254	Organic Acids	↑ ^#^	↓
40	Dopamine	C_8_H_11_NO_2_	00073	Phenols	↑ ^###^	↓ ***
41	Pantothenic acid	C_9_H_17_NO_5_	00210	Vitamin	↑ ^#^	↓

^#^*p* < 0.05, ^##^ *p* < 0.01, ^###^ *p* < 0.001, the model group versus the sham group. * *p* < 0.05, ** *p* < 0.01, *** *p* < 0.001, the GSTTF-treated group versus the model group.

## Supplementary Materials

The following are available online, Figure S1: The typical GC-MS TIC of serum samples, Figure S2: Representative mass spectra of the detected metabolite, Table S1: The detailed information of the selected metabolites.

## References

[1] Strong K., Mathers C., Bonita R. (2007). Preventing stroke: Saving lives around the world. Lancet Neurol..

[2] Kunz A., Iadecola C. (2008). Cerebral vascular dysregulation in the ischemic brain. Handbook Clin. Neurol..

[3] Berkhemer O.A., Fransen P.S., Beumer D., Van Den Berg L.A., Lingsma H.F., Yoo A.J., Schonewille W.J., Vos J.A., Nederkoorn P.J., Wermer M.J. (2015). A randomized trial of intraarterial treatment for acute ischemic stroke. New Engl. J. Med..

[4] Fonarow G.C., Smith E.E., Saver J.L., Reeves M.J., Bhatt D.L., Grau-Sepulveda M.V., Olson D.M., Hernandez A.F., Peterson E.D., Schwamm L.H. (2011). Timeliness of tissue-type plasminogen activator therapy in acute ischemic stroke: Patient characteristics, hospital factors, and outcomes associated with door-to-needle times within 60 min. Circulation.

[5] Chen C., Venketasubramanian N., Gan R.N., Lambert C., Picard D., Chan B.P., Chan E., Bousser M.G., Xuemin S. (2009). Danqi Piantang Jiaonang (DJ), a traditional Chinese medicine, in poststroke recovery. Stroke.

[6] Geng J.-L., Aa J.-Y., Feng S.-Q., Wang S.-Y., Wang P., Zhang Y., Ouyang B.-C., Wang J.-K., Zhu Y.-J., Huang W.-Z. (2017). Exploring the neuroprotective effects of ginkgolides injection in a rodent model of cerebral ischemia–reperfusion injury by GC–MS based metabolomic profiling. J. Pharmaceut. Biomed. Anal..

[7] Liu M., Liu X., Wang H., Xiao H., Jing F., Tang L., Li D., Zhang Y., Wu H., Yang H. (2016). Metabolomics study on the effects of Buchang Naoxintong capsules for treating cerebral ischemia in rats using UPLC-Q/TOF-MS. J. Ethnopharmacol..

[8] Kostova I., Dinchev D. (2005). Saponins in Tribulus terrestris–chemistry and bioactivity. Phytochem. Rev..

[9] Sucher N.J. (2006). Insights from molecular investigations of traditional Chinese herbal stroke medicines: Implications for neuroprotective epilepsy therapy. Epilepsy Behav..

[10] Jiang E.-P., Li H., Chen J.-G., Yang S.-J. (2011). Protection by the gross saponins of Tribulus terrestris against cerebral ischemic injury in rats involves the NF-*κ*B pathway. Acta Pharm. Sinica B.

[11] Wang Y., Liu S., Hu Y., Li P., Wan J.-B. (2015). Current state of the art of mass spectrometry-based metabolomics studies–a review focusing on wide coverage, high throughput and easy identification. RSC Adv..

[12] Sévin D.C., Kuehne A., Zamboni N., Sauer U. (2015). Biological insights through nontargeted metabolomics. Curr. Opin. Biotech..

[13] Zaitsu K., Hayashi Y., Kusano M., Tsuchihashi H., Ishii A. (2016). Application of metabolomics to toxicology of drugs of abuse: A mini review of metabolomics approach to acute and chronic toxicity studies. Drug Metab. Pharmacok..

[14] Van der Worp H.B., van Gijn J. (2007). Acute ischemic stroke. New Engl. J. Med..

[15] Hickey J. (2013). Clinical Practice of Neurological & Neurosurgical Nursing.

[16] Dole V.P. (1956). A relation between non-esterified fatty acids in plasma and the metabolism of glucose. J. Clin. Investig..

[17] Stinkens R., Goossens G.H., Jocken J.W., Blaak E.E. (2015). Targeting fatty acid metabolism to improve glucose metabolism. Obes. Rev..

[18] Rambold A.S., Cohen S., Lippincott-Schwartz J. (2015). Fatty acid trafficking in starved cells: Regulation by lipid droplet lipolysis, autophagy, and mitochondrial fusion dynamics. Dev. Cell.

[19] Lee C.-H., Olson P., Evans R.M. (2003). Minireview: Lipid metabolism, metabolic diseases, and peroxisome proliferator-activated receptors. Endocrinology.

[20] Weisinger H.S., Armitage J.A., Sinclair A.J., Vingrys A.J., Burns P.L., Weisinger R.S. (2001). Perinatal omega-3 fatty acid deficiency affects blood pressure later in life. Nat. Med..

[21] Rizos E.C., Ntzani E.E., Bika E., Kostapanos M.S., Elisaf M.S. (2012). Association between omega-3 fatty acid supplementation and risk of major cardiovascular disease events: A systematic review and meta-analysis. Jama.

[22] Siess W., Scherer B., Böhlig B., Roth P., Kurzmann I., Weber P. (1980). Platelet-membrane fatty acids, platelet aggregation, and thromboxane formation during a mackerel diet. Lancet.

[23] Sanders T., Roshanai F. (1983). The influence of different types of ω3 polyunsaturated fatty acids on blood lipids and platelet function in healthy volunteers. Clin. Sci..

[24] Iso H., Sato S., Umemura U., Kudo M., Koike K., Kitamura A., Imano H., Okamura T., Naito Y., Shimamoto T. (2002). Linoleic acid, other fatty acids, and the risk of stroke. Stroke.

[25] Yamagishi K., Folsom A.R., Steffen L.M., Investigators A.S. (2013). Plasma fatty acid composition and incident ischemic stroke in middle-aged adults: The atherosclerosis risk in communities (ARIC) study. Cerebrovas. Dis..

[26] Tilvis R., Erkinjuntti T., Sulkava R., Färkkilä M., Miettinen T. (1987). Serum lipids and fatty acids in ischemic strokes. Am. Heart J..

[27] Yaemsiri S., Sen S., Tinker L.F., Robinson W.R., Evans R.W., Rosamond W., Wasserthiel-Smoller S., He K. (2013). Serum fatty acids and incidence of ischemic stroke among postmenopausal women. Stroke.

[28] Hu F.B., Manson J.E., Willett W.C. (2001). Types of dietary fat and risk of coronary heart disease: A critical review. J. Am. Coll. Nutr..

[29] Ricci S., Celani M.G., Righetti E., Caruso A., De Medio G., Trovarelli G., Romoli S., Stragliotto E., Spizzichino L., Group U.S. (1997). Fatty acid dietary intake and the risk of ischaemic stroke: A multicentre case-control study. J. Neurol..

[30] Bucalossi A., Mori S. (1972). Fatty acid composition of adipose tissue in ischemic heart disease and stroke. Gerontol. Clin..

[31] Kris-Etherton P.M., Yu S. (1997). Individual fatty acid effects on plasma lipids and lipoproteins: Human studies. Am. J. Clin. Nutr..

[32] Neumar R.W. (2000). Molecular mechanisms of ischemic neuronal injury. Ann. Emerg. Med..

[33] Zhang Z.-X., Gao P.-F., Guo X.-F., Wang H., Zhang H.-S. (2011). 1,3,5,7-Tetramethyl-8-(*N*-hydroxysuccinimidyl butyric ester) difluoroboradiaza-*S*-indacene as a new fluorescent labeling reagent for HPLC determination of amino acid neurotransmitters in the cerebral cortex of mice. Anal. Bioanal. Chem..

[34] Zhang Q., Wang J., Zhang C., Liao S., Li P., Xu D., Lv Y., Yang M., Kong L. (2016). The components of Huang-Lian-Jie-Du-Decoction act synergistically to exert protective effects in a rat ischemic stroke model. Oncotarget.

[35] Su L., Zhao H., Zhang X., Lou Z., Dong X. (2016). UHPLC-Q-TOF-MS based serum metabonomics revealed the metabolic perturbations of ischemic stroke and the protective effect of RKIP in rat models. Mol. BioSyst..

[36] Bie X.-D., Chen Y.-Q., Han J., Dai H.-B., Wan H.-T., Zhao T.-F. (2007). Effects of gastrodin on amino acids after cerebral ischemia-reperfusion injury in rat striatum. Asia Pac. J. Clin. Nutr..

[37] Pettigrew L.C., Bang H., Chambless L.E., Howard V.J., Toole J.F., Investigators V. (2008). Assessment of pre-and post-methionine load homocysteine for prediction of recurrent stroke and coronary artery disease in the Vitamin Intervention for Stroke Prevention Trial. Atherosclerosis.

[38] Chiang T., Messing R.O., Chou W.-H. (2011). Mouse model of middle cerebral artery occlusion. J. Visual. Exper..

[39] Longa E.Z., Weinstein P.R., Carlson S., Cummins R. (1989). Reversible middle cerebral artery occlusion without craniectomy in rats. Stroke.

[40] Kind T., Wohlgemuth G., Lee D.Y., Lu Y., Palazoqlu M., Shahbaz S., Fiehn O. (2009). FiehnLib: Mass spectral and retention index libraries for metabolomics based on quadrupole and time-of-flight gas chromatography/mass spectrometry. Anal. Chem..

[41] Chen Q., Lu X., Guo X., Guo Q., Li D. (2017). Metabolomics characterization of two Apocynaceae plants, Catharanthus roseus and Vinca minor, using GC-MS and LC-MS methods in combination. Molecules.

[42] Qiu Y., Cai G., Su M., Chen T., Zheng X., Xu Y., Ni Y., Zhao A., Xu L.X., Cai S., Jia W. (2009). Serum metabolite profiling of human colorectal cancer using GC-TOFMS and UPLC-QTOFMS. J. Proteome Res..

[43] Jiang W., Qiu Y., Ni Y., Su M., Jia W., Du X. (2010). An automated data analysis pipeline for GC-TOF-MS metabonomics studies. J. Proteome Res..

[44] Ni Y., Qiu Y., Jiang W., Suttlemyre K., Su M., Zhang W., Jia W., Du X. (2012). ADAP-GC 2.0: Deconvolution of coeluting metabolites from GC/TOF-MS data for metabolomics studies. Anal. Chem..

[45] Ni Y., Su M., Qiu Y., Jia W., Du X. (2016). ADAP-GC 3.0: Improved peak detection and deconvolution of co-eluting metabolites from GC/TOF-MS data for metabolomics studies. Anal. Chem..

