# Supplementary material for: GC-MS-Based Metabolomics to Reveal the Protective Effect of Gross Saponins of Tribulus terrestris Fruit against Ischemic Stroke in Rat
Source: Molecules. 2019 Feb 22;24(4):793. doi: 10.3390/molecules24040793 (PMC6412276; doi:10.3390/molecules24040793)
Supplement: Supplementary file 1 [file molecules-24-00793-s001.pdf]

Supplementary Materials

Article

GC-MS-Based Metabolomics to Reveal the Protective Effect of Gross Saponins of *Tribulus terrestris* Fruit against Ischemic Stroke in Rat

Yang Wang [1], Hongyu Zhao [2], Yue Liu [2], Wenjun Guo [2], Yanru Bao [2], Manqi Zhang [2], Tunhai Xu [3,*], Shengxu Xie [2], Xinyu Liu [2] and Yajuan Xu [2,*]

[1] Jilin Ginseng Academy, Changchun University of Chinese Medicine, Changchun, Jilin 130117, China; white-wing@163.com
[2] Key Laboratory of Medicinal Materials, Jilin Academy of Chinese Medicine Sciences, Changchun, Jilin 130021, China; fixiov5815@126.com (H.Z.); yue_liu_tryh@126.com (Y.L.); 19969540912@163.com (W.G.); 024baoyanru@163.com (Y.B.); mzhan016@fiu.edu (M.Z.); jshx222@126.com (S.X.); liuxinyu6868@126.com (X.L.)
[3] Academy of Traditional Chinese Medicine, Beijing University of Chinese Medicine, Beijing 100029, China
* Correspondence: thxu@163.com (T.X.); xyj6492@126.com (Y.X.); Tel./Fax.: +86-431-8605-8628 (Y.X.)

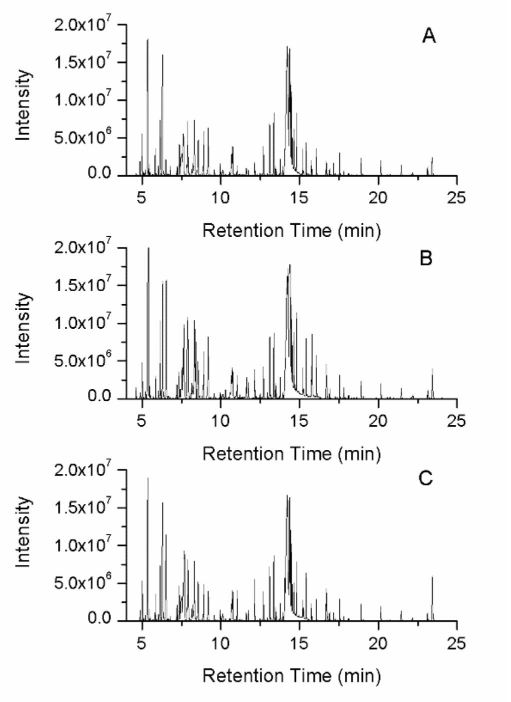

Figure S1 The typical GC-MS total ion chromatograms (TICs) of MCAO rat serum samples, (A) sham group, (B) model group and (C) GSTTF-treated group.

Dodecanoic acid

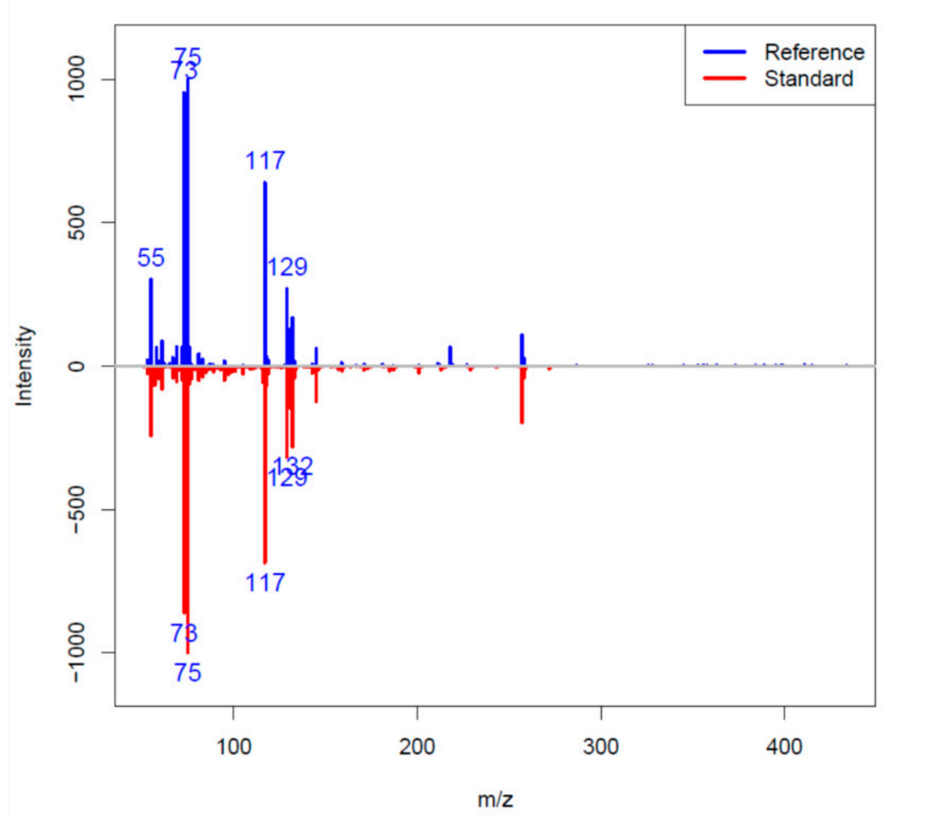

Heptadecanoic acid

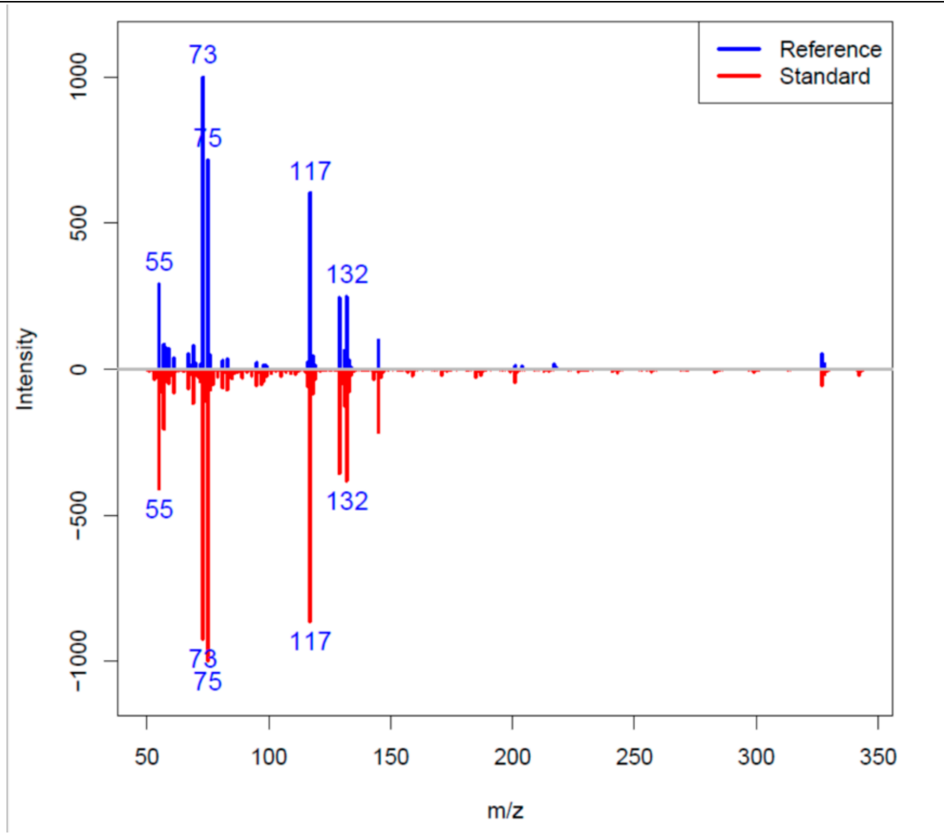

Palmitic acid

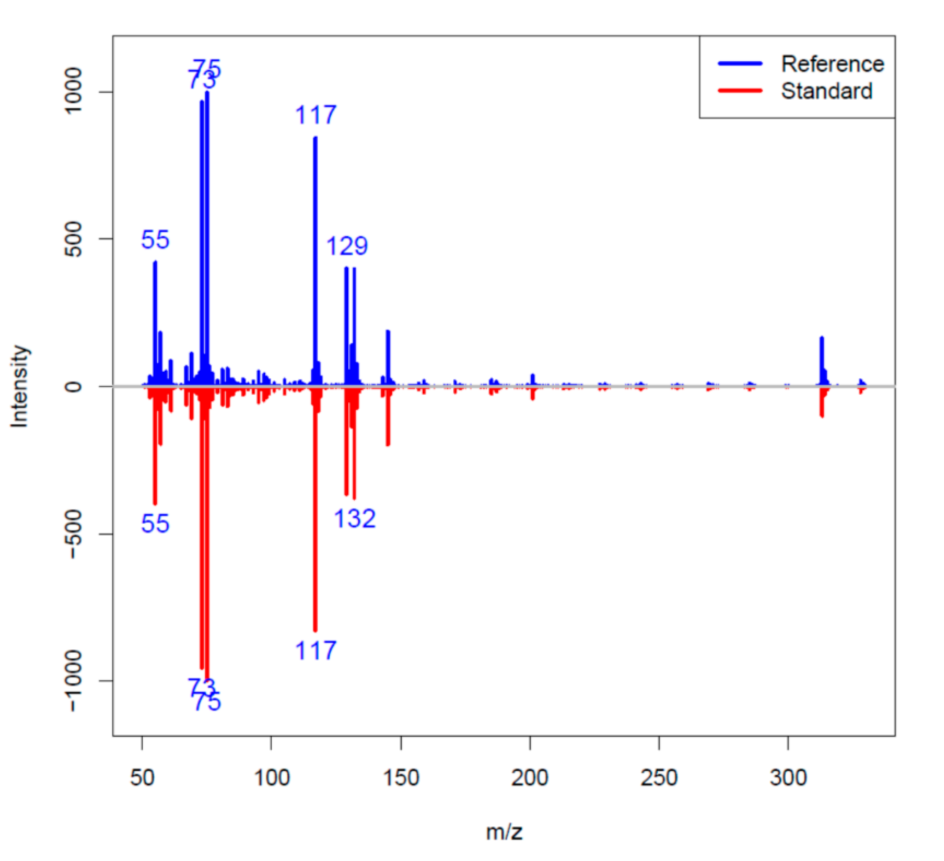

Stearic acid

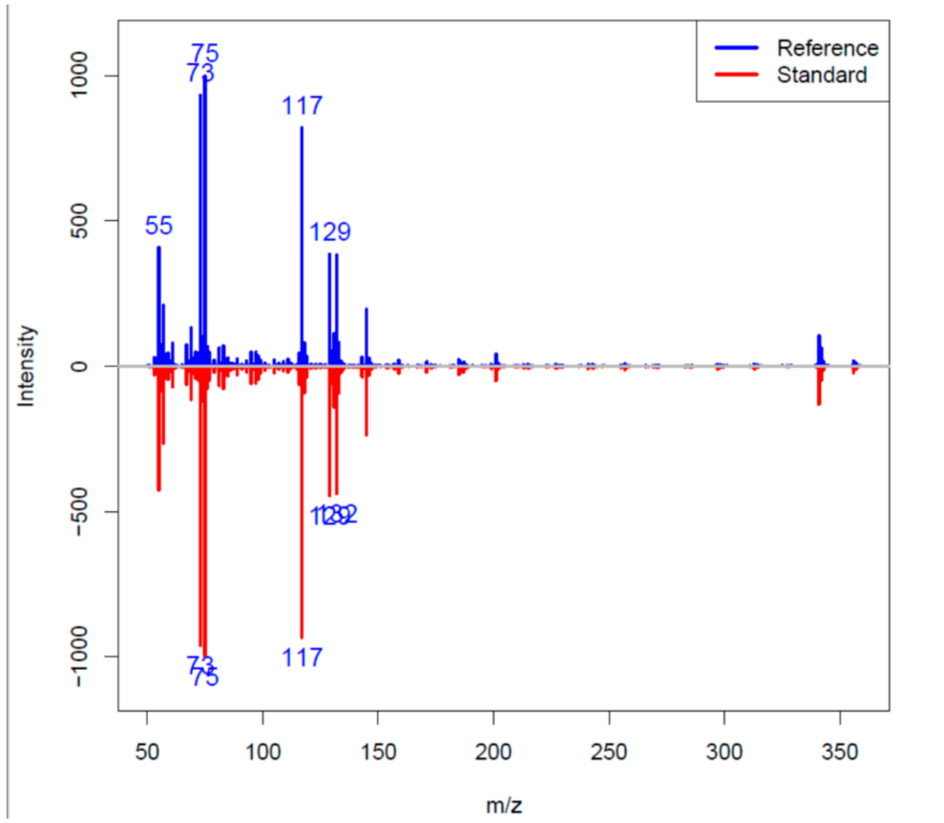

Docosahexaenoic acid

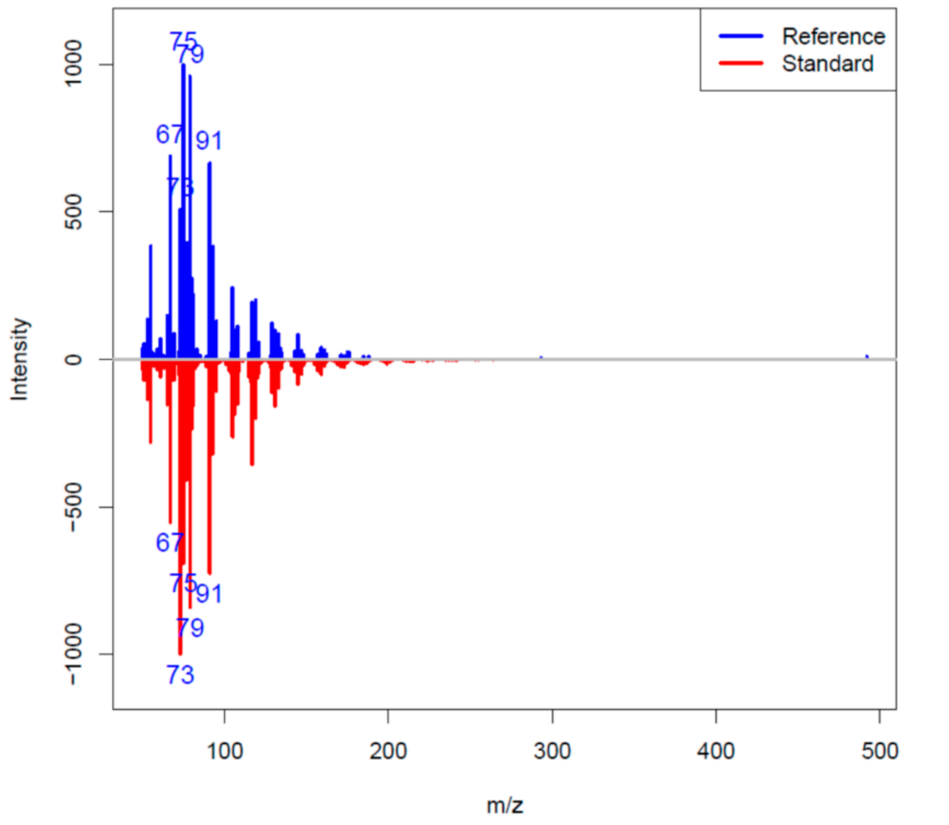

Glucose

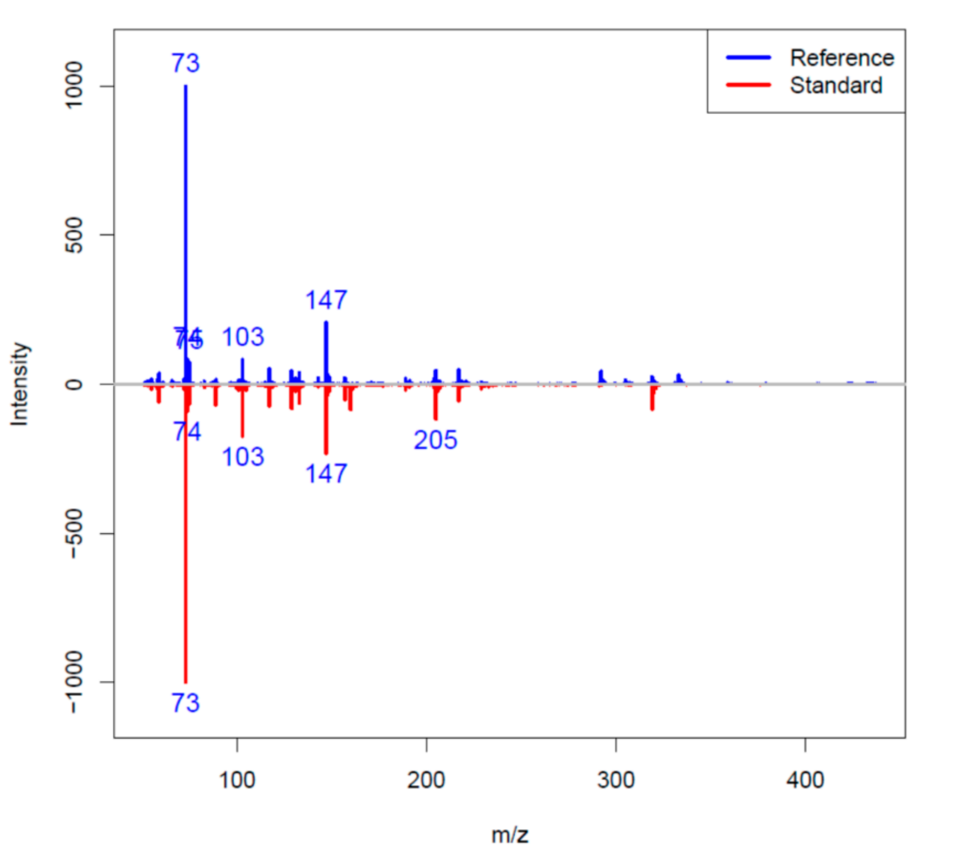

Isoleucine

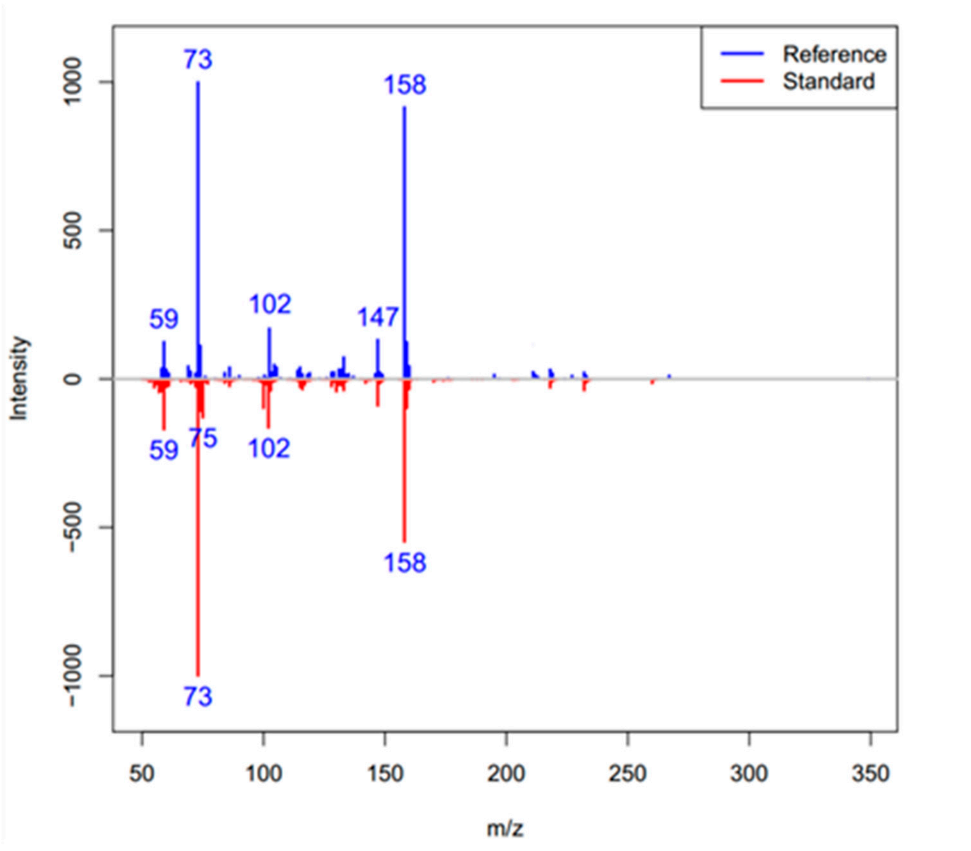

Figure S2 Representative mass spectra of the detected metabolite

Table S1 The retention index and fragment ions of selected trimethylsilylated metabolites.

Name	Retention index	m/z of fragment ions
**Caproic acid**	340436	73,75,117,173,
**Isoleucine**	501137	59,73,75,158
**Picolinic acid**	523447	51,73,78,180,136
**Succinic acid**	523758	55,73,75,147
**Serine**	558165	73,75,100,204
**Pipecolic acid**	563977	73,156,157,158,230
**Beta-Alanine**	592771	59,73,86,174
**Malic acid**	640508	55,73,75,147
**Creatine**	664999	73,100,115,143,171
**4-Hydroxy-proline**	668459	73,75,140,230,
**Creatinine**	686201	73,100,115,143
**Dodecanoic acid**	729672	73,75,117,257
**Xylose**	739997	73,103,147,217,307
**Homocysteine**	743844	73,75,128,234
**Ribonolactone**	756235	73,102,117,133,217
**Ribose**	761779	73,75,103,147
**Arabitol**	764105	73,75,103,147
**O-Phosphoethanolamine**	799503	73,100,172,174,299
**Myristoleic acid**	829881	73,75,89,117
**Ornithine**	832463	70,73,74,142
**Myristic acid**	840005	73,75,117,174
**Methionine sulfoxide**	851360	73,100,128,140,301
**Pentadecanoic acid**	885806	73,75,117
**Glucose**	891963	73,147,160,205
**Pantothenic acid**	905323	73,98,103,260,363
**Palmitoleic acid**	923365	55,73,75,117
**Palmitic acid**	933113	73,75,117
**Allantoic acid**	944298	73,100,147,188,331
**Dopamine**	960330	73,179,267,268,269
**Heptadecanoic acid**	978133	73,75,117
**Linoleic acid**	1011058	55,67,73,75
**Petroselinic acid**	1013794	73,75,83,117,129
**Oleic acid**	1013809	55,73,75,117
**Stearic acid**	1021987	73,75,117
**Arachidonic acid**	1077225	67,73,75,79
**Glucose 6-phosphate**	1080958	73,129,147,160,387
**Docosahexaenoic acid**	1157484	67,73,75,79,91
**Inosine**	1173808	73,75,103,217
**Guanosine**	1246885	73,74,75,103
**Pregnenolone**	1278959	73,87,91,93,100
**Cholesterol**	1413030	73,75,129
